# Supplementary material for: Revealing disorder parameter and deformation electron density using electron diffraction
Source: Nat Commun. 2025 Jul 1;16:5811. doi: 10.1038/s41467-025-60966-0 (PMC12214857; doi:10.1038/s41467-025-60966-0)
Supplement: Supplementary file 1 — Supplementary Information [file 41467_2025_60966_MOESM1_ESM.pdf]

Supplementary information for

Revealing disorder parameter and deformation electron density using electron diffraction

Weixiao Lin[1,2,6], Zefan Xue[1,2,6], Wenjun Cui[1,2], Andreas Kulovits[3], Hao Ren[4], Wen Zhao[4], Jinsong Wu[1,2], Gustaaf Van Tendeloo[2,5], Jörg Wiezorek[3], Xiahan Sang[1,2,]*

[1] State Key Laboratory of Advanced Technology for Materials Synthesis and Processing, Wuhan University of Technology, Wuhan 430070, China

[2] Nanostructure Research Center, Wuhan University of Technology, Wuhan 430070, China

[3] Department of Mechanical Engineering and Materials Science, University of Pittsburgh, 636 Benedum Hall, 3700 O'Hara Street, PA 15261, USA

[4] School of Materials Science and Engineering, China University of Petroleum (East China), Qingdao 266580, China

[5] EMAT (Electron Microscopy for Materials Science), University of Antwerp, Groenenborgerlaan 171, Antwerp 2020, Belgium

[6] These authors contributed equally

*Corresponding author: xhsang@whut.edu.cn

Supplementary Note 1 The relationship between disorder parameter η and the conventional definition of long-range order parameter S

The disorder parameter defined here is based on a conventional definition of long-range order parameters S[1]. If we use tP2 cell as an example, the long-range order parameter of Fe at (0 0 0) is defined as $S = \frac{Occ.(\mathrm{Fe_{Fe}}) - F_{\mathrm{Fe}}}{1 - F_{\mathrm{Fe}}}$, where F_{Fe} = 0.5 is the atomic fraction of Fe in FePd, and $Occ.(\mathrm{Fe_{Fe}})$ is the occupancy of Fe at (0 0 0), which is related to $Occ.(\mathrm{Fe_{Pd}})$ as follows,

$$Occ.(\mathrm{Fe_{Fe}}) + Occ.(\mathrm{Fe_{Pd}}) = 1$$

Therefore, $S = 1 - 2 * Occ.(\mathrm{Fe_{Pd}}) = 1 - \eta$.

Supplementary Note 2 Structure factors of tP2 FePd

There are four atoms in the tP2 cell: Pd atom at (0 0 0) with occupancy $\frac{\eta}{2}$; Pd atom at (0.5 0.5 0.5) with occupancy $1 - \frac{\eta}{2}$; Fe atom at (0 0 0) with occupancy $1 - \frac{\eta}{2}$; Fe atom at (0.5 0.5 0.5) with occupancy $\frac{\eta}{2}$;

$$F_g^X = \sum_i f_i(s) e^{-B_i s^2} e^{-2\pi i g \cdot r_i}$$

$$= [\left(1 - \frac{\eta}{2}\right) f_{\mathrm{Fe}}(s) e^{-B_{\mathrm{Fe}} s^2} + \frac{\eta}{2} f_{\mathrm{Pd}}(s) e^{-B_{\mathrm{Pd}} s^2}] e^{-2\pi i (h \cdot 0 + k \cdot 0 + l \cdot 0)} + [\frac{\eta}{2} f_{\mathrm{Fe}}(s) e^{-B_{\mathrm{Fe}} s^2} + \left(1 - \frac{\eta}{2}\right) f_{\mathrm{Pd}}(s) e^{-B_{\mathrm{Pd}} s^2}] e^{-2\pi i (h \cdot 0.5 + k \cdot 0.5 + l \cdot 0.5)}$$

$$= \left(1 - \frac{\eta}{2}\right) f_{\mathrm{Fe}}(s) e^{-B_{\mathrm{Fe}} s^2} + \frac{\eta}{2} f_{\mathrm{Pd}}(s) e^{-B_{\mathrm{Pd}} s^2} + [\frac{\eta}{2} f_{\mathrm{Fe}}(s) e^{-B_{\mathrm{Fe}} s^2} + \left(1 - \frac{\eta}{2}\right) f_{\mathrm{Pd}}(s) e^{-B_{\mathrm{Pd}} s^2}] (-1)^{h+k+l}$$

when $\mathrm{h + k + l}$ is odd:

$$F_g^X = \left(1-\frac{\eta}{2}\right) f_{\mathrm{Fe}}(s)e^{-B_{\mathrm{Fe}}s^2} + \frac{\eta}{2} f_{\mathrm{Pd}}(s)e^{-B_{\mathrm{Pd}}s^2} - \frac{\eta}{2} f_{\mathrm{Fe}}(s)e^{-B_{\mathrm{Fe}}s^2} - \left(1-\frac{\eta}{2}\right) f_{\mathrm{Pd}}(s)e^{-B_{\mathrm{Pd}}s^2}$$
$$= (1-\eta)\left(f_{\mathrm{Fe}}(s)e^{-B_{\mathrm{Fe}}s^2} - f_{\mathrm{Pd}}(s)e^{-B_{\mathrm{Pd}}s^2}\right)$$

when $\mathrm{h+k+l}$ is even:

$$F_g^X = \left(1-\frac{\eta}{2}\right) f_{\mathrm{Fe}}(s)e^{-B_{\mathrm{Fe}}s^2} + \frac{\eta}{2} f_{\mathrm{Pd}}(s)e^{-B_{\mathrm{Pd}}s^2} + \frac{\eta}{2} f_{\mathrm{Fe}}(s)e^{-B_{\mathrm{Fe}}s^2} + \left(1-\frac{\eta}{2}\right) f_{\mathrm{Pd}}(s)e^{-B_{\mathrm{Pd}}s^2}$$
$$= f_{\mathrm{Fe}}(s)e^{-B_{\mathrm{Fe}}s^2} + f_{\mathrm{Pd}}(s)e^{-B_{\mathrm{Pd}}s^2}$$

Supplementary Note 3 The three analytical point spread function (PSF) models: Gaussian distribution, Lorentzian distribution, and Voigt distribution

Three analytical PSF models were used in the refinements: a Gaussian distribution $G(x;\sigma)$, a Lorentzian distribution $L(x;\gamma)$, and their convolution, the Voigt distribution $V(x;\sigma;\gamma)$. These are defined as:

$$G(x;\sigma) \equiv \frac{e^{-\frac{x^2}{2\sigma^2}}}{\sqrt{2\pi}\sigma}$$

$$L(x;\gamma) \equiv \frac{\gamma}{\pi(\gamma^2+x^2)}$$

$$V(x;\sigma;\gamma) \equiv \int_{-\infty}^{\infty} G(x';\sigma)L(x-x';\gamma)dx'$$

The full width at half maximum (FWHM) for the three distributions are:

Gaussian distribution: $f_G = 2\sqrt{2\ln(2)}\sigma$;

Lorentzian distribution: $f_L = 2\gamma$;

Voigt distribution[2]: $f_V = 0.5343 \times f_L + \sqrt{0.2169 f_L^2 + f_G^2}$.

Supplementary Note 4 Estimation of energy transferred from the electron beam to FePd and the threshold energy

The maximum elastic collision energies (E_{max}) transferred from the electron beam to an atom can be calculated using the following equation[3, 4]:

$$E_{\mathrm{max}} = \frac{2ME(E+2mc^2)}{(M+m)^2c^2+2ME}$$

where M and m refer to the mass of the atom and the rest electron mass (9.11 × 10^{-31} kg), respectively. E is the incident electron energy and c is the speed of light. The table below summarizes E_{max} for Fe and Pd at 200 kV and 300 kV.

Maximum energy E_{max} transferred from the electron beam to Fe and Pd atoms.

	M	200 kV	300 kV
Fe	9.27 × 10^{-26} kg	9.40 eV	15.25 eV
Pd	1.77 × 10^{-25} kg	4.93 eV	8.00 eV

The threshold energies (E_d) of Pd and Pd metals have been measured to be 20 eV and 34 eV, respectively[5, 6]. Experimental E_d for FePd has not been reported. However, it has been reported that E_d is roughly related to the melting temperature T_m by the equation E_d = 0.0076 × T_m + 9.8441. Using FePd T_m = 1577.15 K in the phase diagram[7], E_d is estimated to be 22.83 eV. All these values are larger than the calculated E_{max}, confirming that the electron beam causes negligible beam damage.

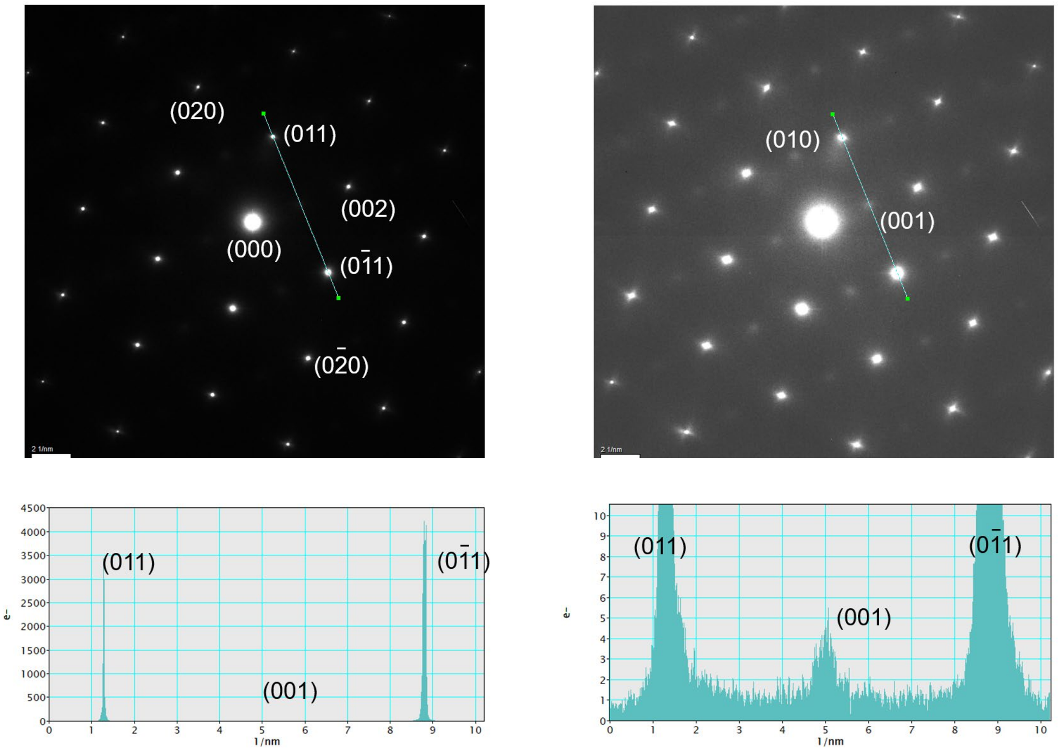

Supplementary Fig. 1 A typical selected area electron diffraction pattern acquired along [100] zone axis of a tP2 cell (or [110] zone axis of disordered FCC FePd). The left part is the diffraction pattern with normal contrast, and the right has enhanced contrast to show the weak 'ordered' reflections due to tP2 ordering. The line profile shows that main reflections such as (011) spots have intensities around 4000, while the (001) spot has an intensity of around 4, which is only 0.1% of the main reflections. All the weak diffraction spots are from tP2 ordering, indicating that any other long-range ordering should be negligibly weak.

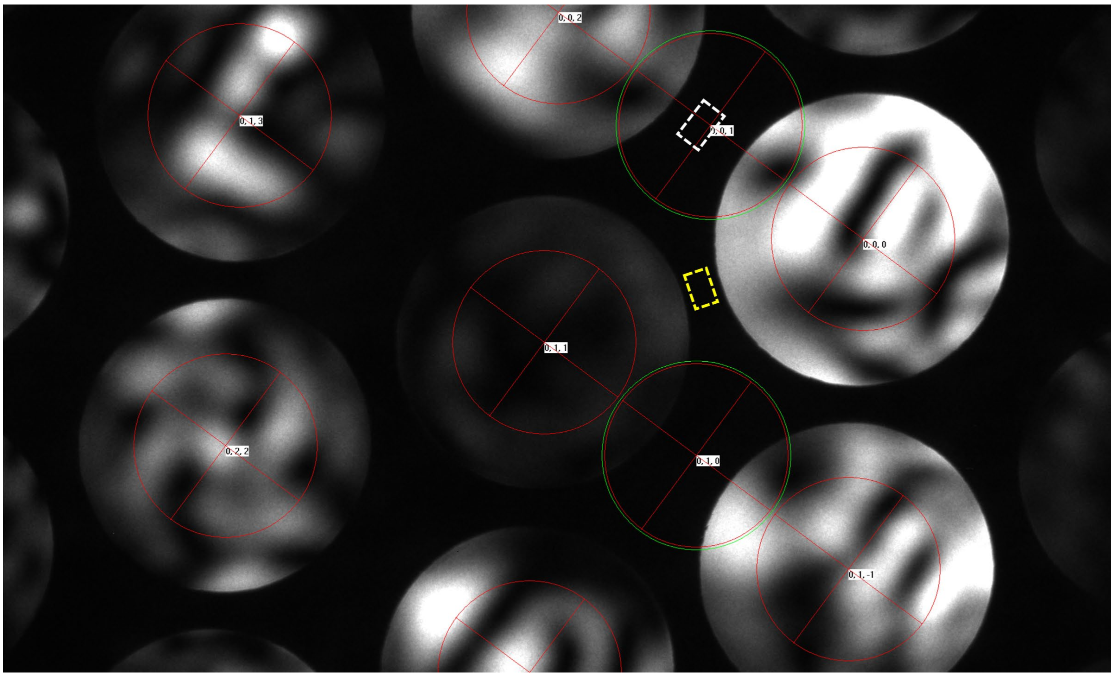

Supplementary Fig. 2 Comparison between the average intensity from the (001) disc and the background of the CBED pattern shown in Fig. 2c. The averaged intensity from the white dashed rectangle region of the (001) disc is 66, while the background intensity from the yellow dashed rectangle region is 70, proving that the (001) disc has intensity comparable to the background.

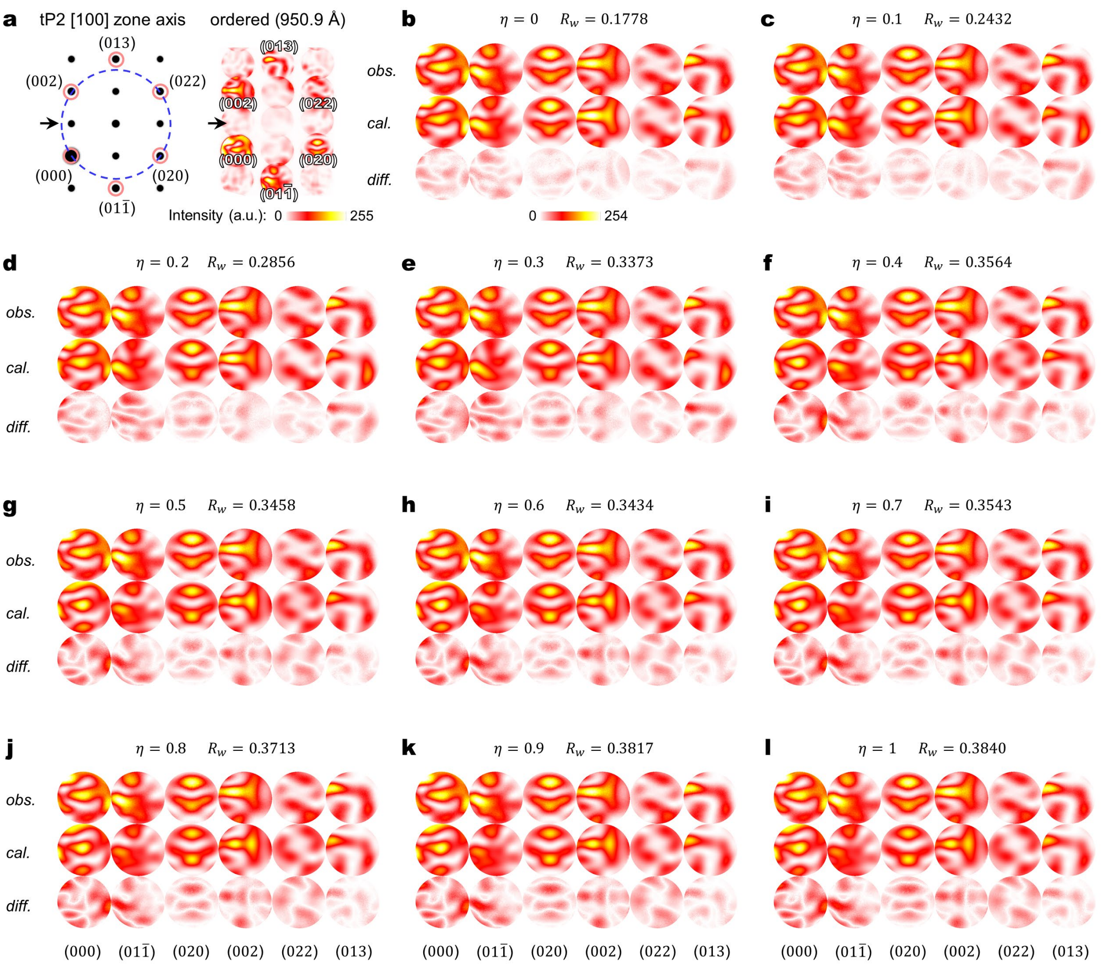

Supplementary Fig. 3 QCBED refinement results for the CBED pattern in Fig. 2b using different η. a Simulated SAED pattern of tP2 FePd along the [100] zone axis. (020), (002), and (022) discs are precisely on the Ewald sphere surface for this MBOZA condition. The blue dashed circle is the intersection between the Ewald sphere and ZOLZ. **b-l** QCBED refinement results showing the observed (*obs.*), calculated (*cal.*), and difference (*diff.*, defined as |*obs.* - *cal.*|) intensity distribution of the six diffraction discs (000), (01$\bar{1}$), (020), (002), (022) and (013) for different η. The $(0kl)$-type disks with $k + l$ odd are indicated by black arrows. Images b-l use the same color scale range.

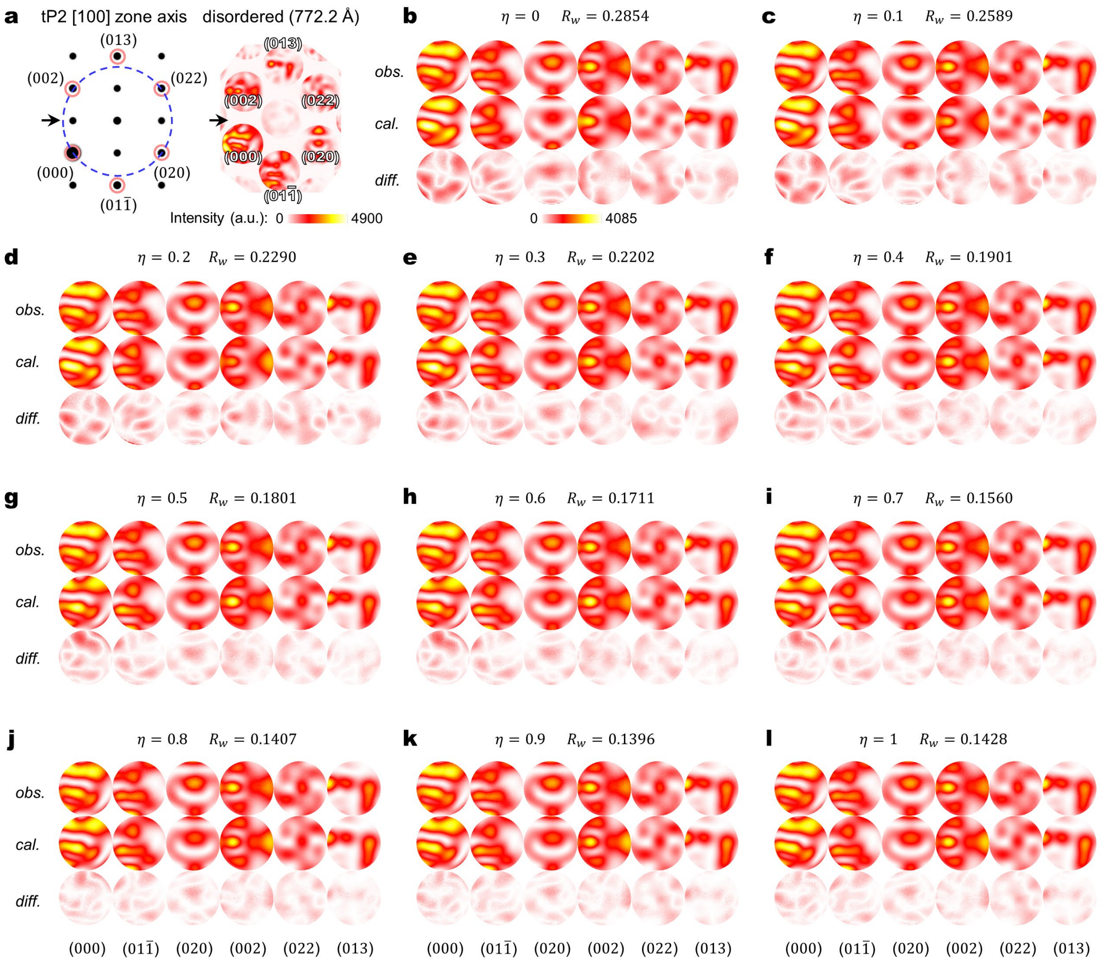

Supplementary Fig. 4 QCBED refinement results for the CBED pattern in Fig. 2c using different $\boldsymbol{\eta}$**. a** Simulated SAED pattern of tP2 FePd along the [100] zone axis. (020), (002), and (022) discs are precisely on the Ewald sphere surface for this MBOZA condition. The blue dashed circle is the intersection between the Ewald sphere and ZOLZ. **b-l** QCBED refinement results showing the observed (*obs.*), calculated (*cal.*), and difference (*diff.*, defined as |*obs.* - *cal.*|) intensity distribution of the six diffraction discs (000), (01$\bar{1}$), (020), (002), (022) and (013) for different η. The supposed locations of ($0kl$)-type disks with $k + l$ odd are indicated by black arrows. Images b-l use the same color scale range.

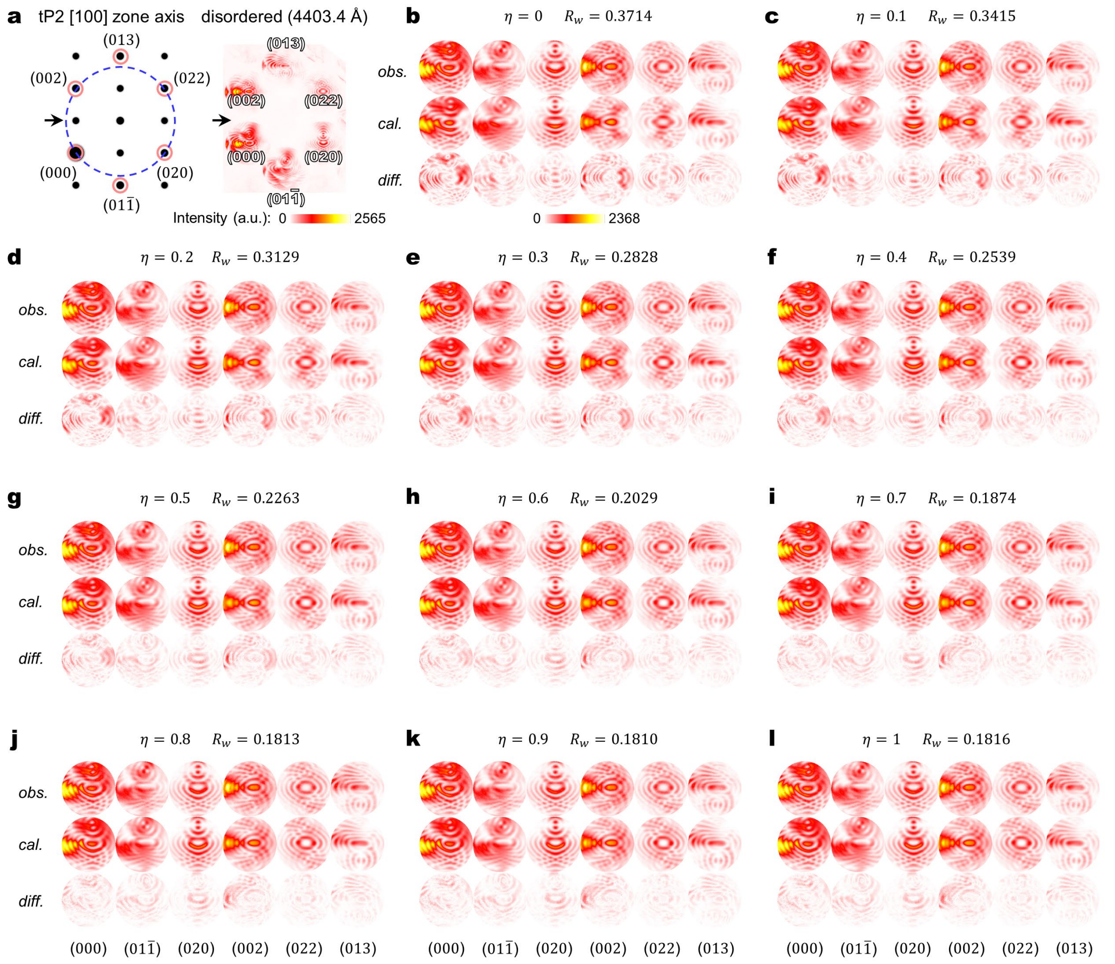

Supplementary Fig. 5 QCBED refinement results for the CBED pattern in Fig. 2d using different η. a Simulated SAED pattern of tP2 FePd along the [100] zone axis. (020), (002), and (022) discs are precisely on the Ewald sphere surface for this MBOZA condition. The blue dashed circle is the intersection between the Ewald sphere and ZOLZ. **b-l** QCBED refinement results showing the observed (*obs.*), calculated (*cal.*), and difference (*diff.*, defined as |*obs.* - *cal.*|) intensity distribution of the six diffraction discs (000), (01$\bar{1}$), (020), (002), (022) and (013) for different η. The supposed locations of $(0kl)$-type disks with $k + l$ odd are indicated by black arrows. Images b-l use the same color scale range.

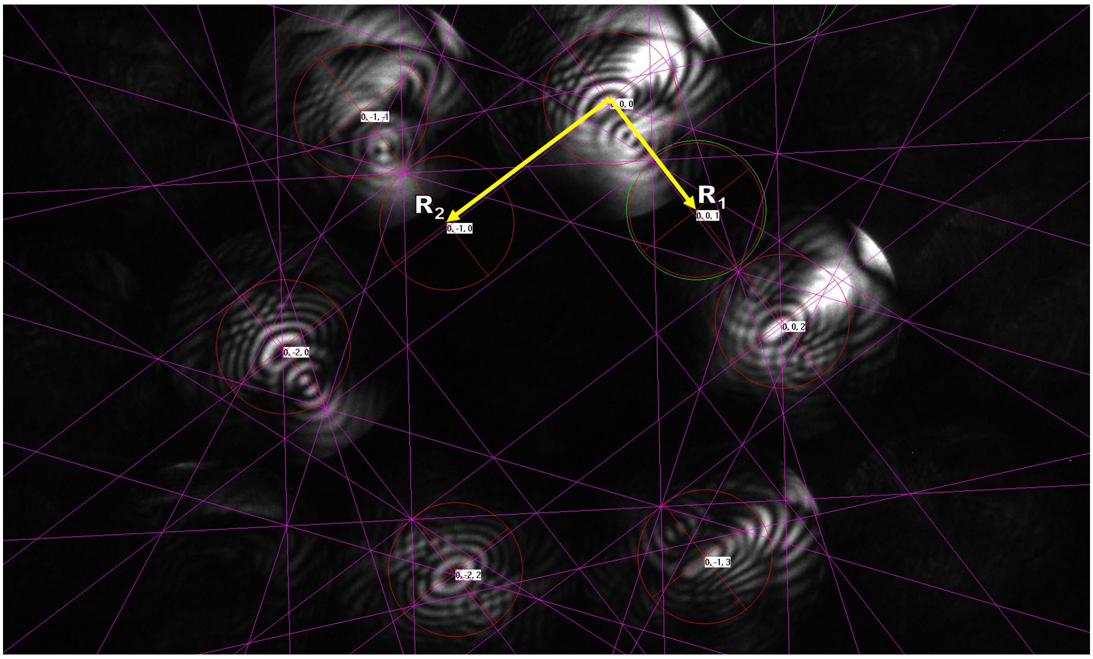

Supplementary Fig. 6 Geometric distortion measurement using the AdjCbd software. The AdjCbd interface shows the locations of discs and Kikuchi lines. The two nearest vectors, R_1 and R_2, are indicated by yellow arrows. The locations of the center disc, the two nearest discs, R_1 and R_2, and the Kikuchi pattern can be freely adjusted. Here, the mirror symmetries in the $(0\bar{2}0)$ and (002) discs, and the 2-fold symmetry in the $(0\bar{2}2)$ disc can be used to guide the location of discs and Kikuchi lines. The angle and ratio between R_2 and R_1 are then compared with the standard lattice constant to evaluate the linear distortion. For this particular CBED pattern, R_2/R_1 is 1.459, and the angle is 89.5°. Using the standard lattice constants, a = 2.692 Å and c = 3.807 Å, the ratio should be 1.414, and the angle should be 90°. The linear distortion is therefore determined by restoring the ratio and angle to the desired values.

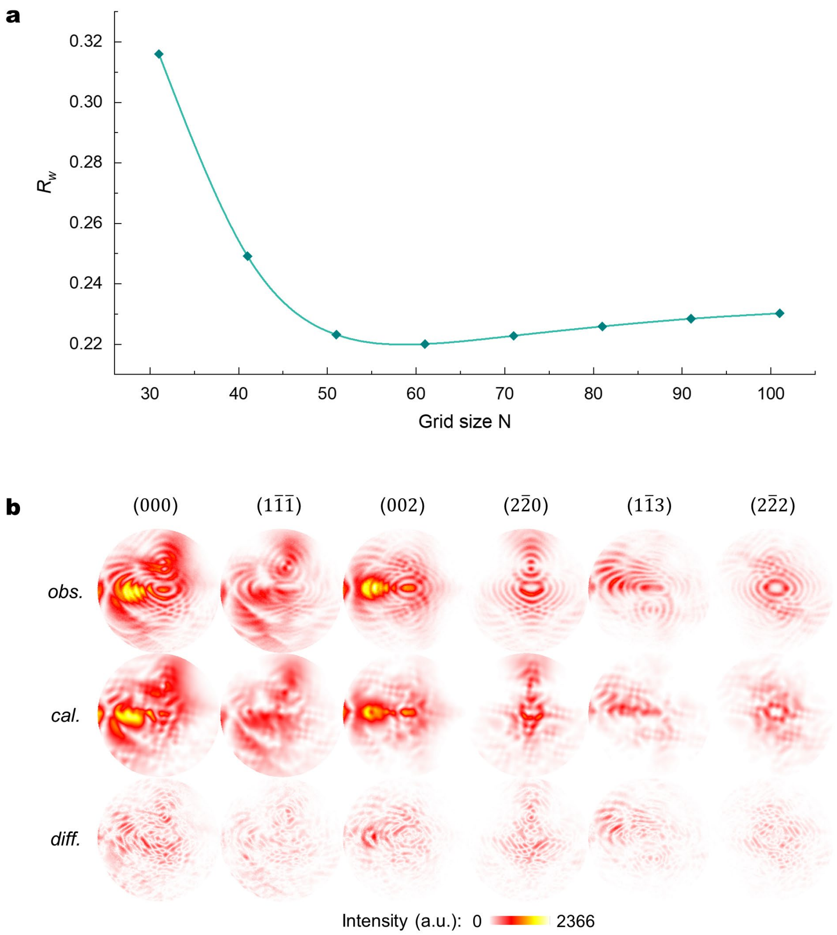

Supplementary Fig. 7 The influence of grid size N on R_w value. From the excited-row method to the MBOZA method, the refined data points change from 1D to 2D, and a big challenge is the computation time. It is very time-consuming to calculate the diffraction intensity of every pixel by solving the Schrodinger equation in the periodic potential with a different beam-sample orientation. A conventional approach is to simulate data points for a N×N grid, and then interpolate the data points in between the grid points. The number N can be determined by performing QCBED refinement using different N and then checking which number yields the lowest R_w value. As N changes from 31 to 101, R_w first decreases, reaches a minimum for N = 61, and then slowly increases again. For N = 31, the grid size is too small, and the calculation is significantly under-sampling, resulting in very large R_w. As N further increases beyond 61, the computation time scales with N squared, but R_w did not improve. Therefore, N = 61 is a good compromise between time and accuracy for QCBED refinement.

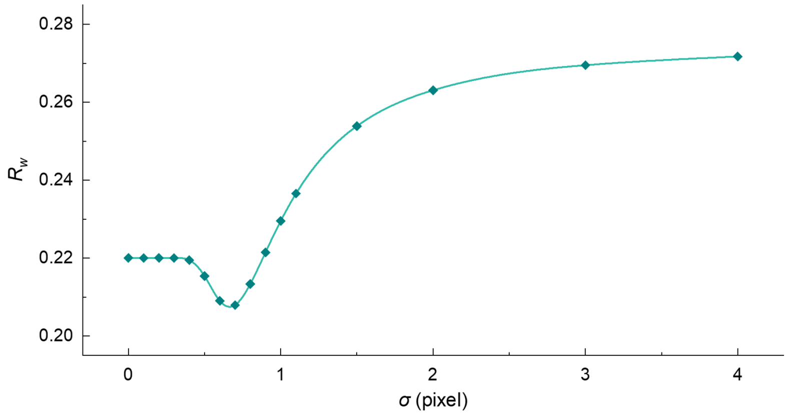

Supplementary Fig. 8 The influence of Gaussian convolution on R_w for grid size N = 61. The overall blurring effect is considered by convoluting the calculated CBED pattern using 2D Gaussian distribution with different σ. For grid size N = 61, as σ increases, R_w first decreases and then increases.

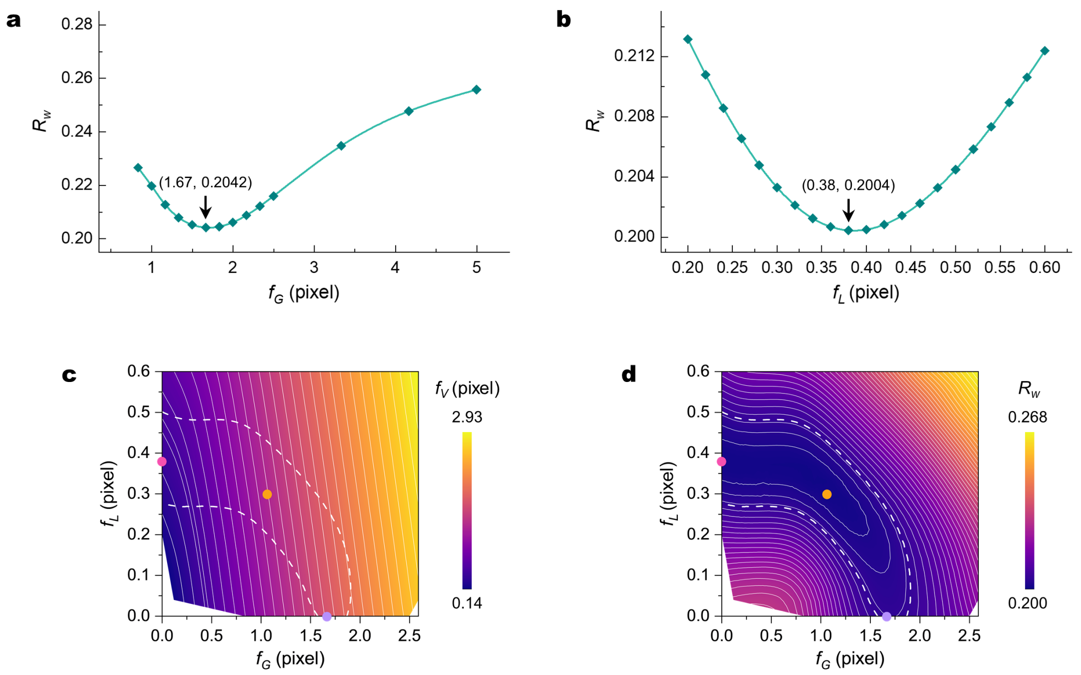

Supplementary Fig. 9 Refinement results using three analytical PSF models (grid size 101). (**a**) R_w value as a function of Gaussian FWHM (f_G). The minimum R_w of 0.2042 is achieved at f_G = 1.67 pixels. (**b**) Lorentzian FWHM (f_L) used in the PSF convolution. The minimum R_w is slightly lower at 0.2004, achieved at f_L = 0.38 pixels. **(c-d)** Optimization of Voigt PSF parameters. Contour map shows R_w value as a function of the Gaussian FWHM f_G and Lorentzian FWHM f_L components of the Voigt profile used for convolution. The global minimum R_w = 0.2002 was found using a Voigt profile with f_G = 1.06 pixels and f_L = 0.3 pixels. This represents only a marginal improvement over the pure Lorentzian fit. The contour map shows a relatively flat minimum, indicating that similar R_w values are obtained for a range of f_G and f_L combinations. The refined structure factors (F_{111} and F_{200}) varied by only ~0.003 e$^-$/atom across the three PSF models, corresponding to a negligible difference of ~0.01%. Other parameters like thickness and the DWF also showed minimal variation.

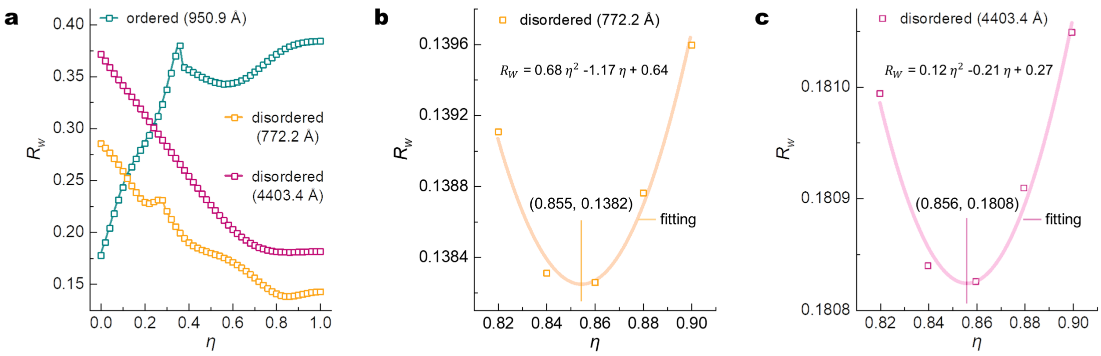

Supplementary Fig. 10 The fitting of η using R_w - η curve. The optimized η can be obtained by fitting the R_w - η curve using quadratic polynomial. The step size for η is 0.02. R_w minimum is obtained for η = 0.855 and 0.856, for disordered CBED patterns with thicknesses being 772.2 and 4403.4 Å, respectively. The orange line (image b) and pink line (image c) represent quadratic polynomial fits to the data, with the respective equation $R_W = 0.68\eta^2 - 1.17\eta + 0.64$ and $R_W = 0.12\eta^2 - 0.21\eta + 0.27$.

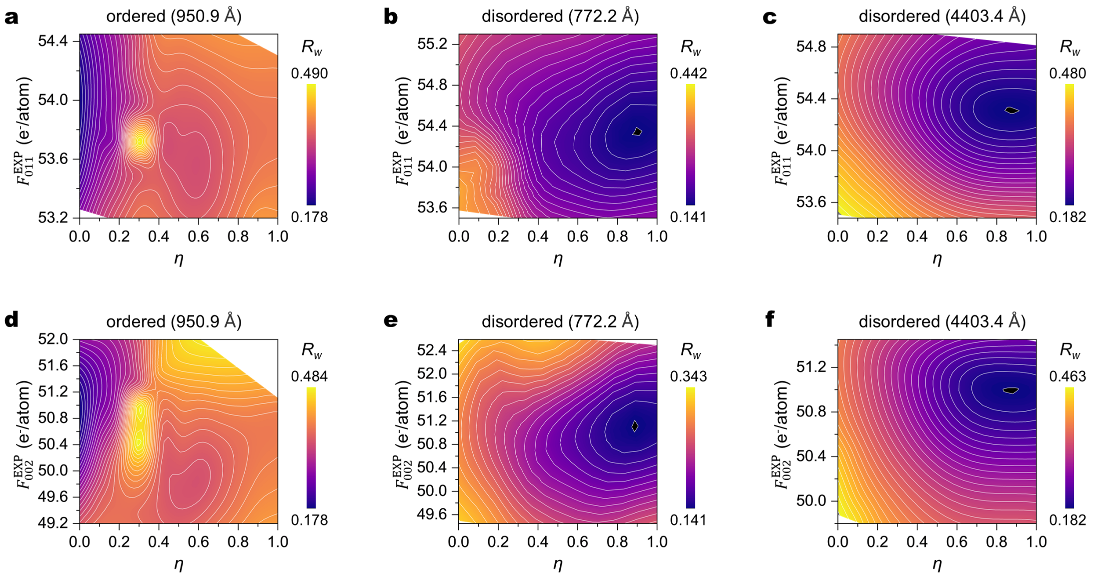

Supplementary Fig. 11 The R_w isosurface of η - F_{011}, η - F_{002} of the three CBED patterns in Fig. 2b-d. Well-defined global minima are observed for all the cases.

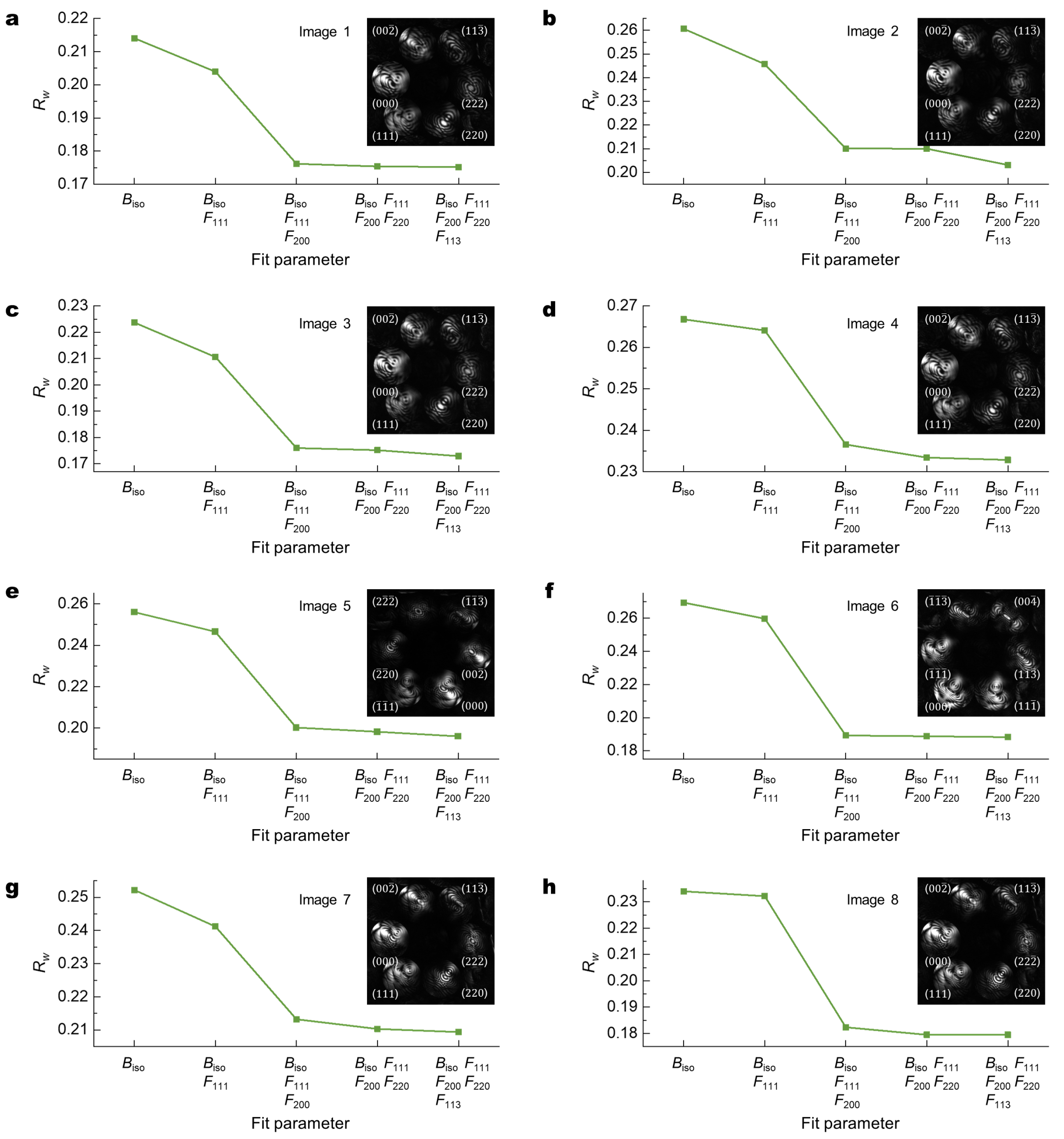

Supplementary Fig. 12 The change of R_w as more structure factors are included in QCBED refinement. As more structure factors are included in the refinement, R_w reaches a plateau for all eight CBED patterns. The first refinement was performed by only relaxing B_{iso}. Then, the first low-order structure factor F_{111} is included in the refinement, which reduces R_w by around 0.01. The inclusion of F_{200} further reduces R_w by around 0.06. After that, including F_{220} and F_{113} in the refinement leads to a negligible decrease of R_w.

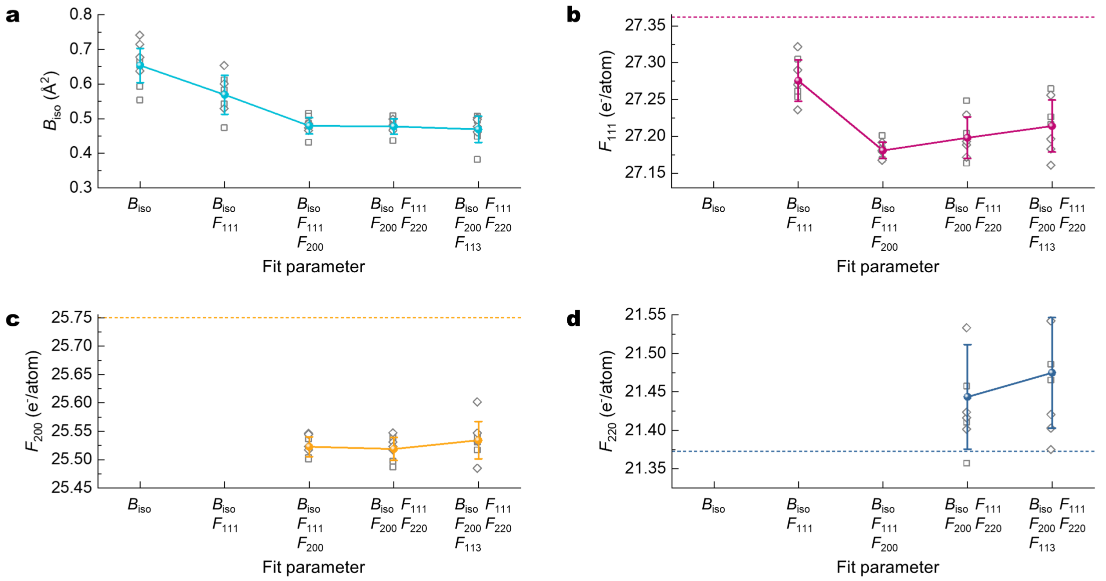

Supplementary Fig. 13 Fitting results of 8 CBED patterns as more structure factors are included in QCBED refinement. When F_{113} is included, the error bars for B_{iso}, F_{111}, and F_{200} increase, but the averaged values do not change much. Adding one parameter to the refinement process encourages the other parameters to vary more freely, which makes it more difficult to find a global minimum. The error bars represent the standard deviation calculated from 8 independent measurements for each data point.

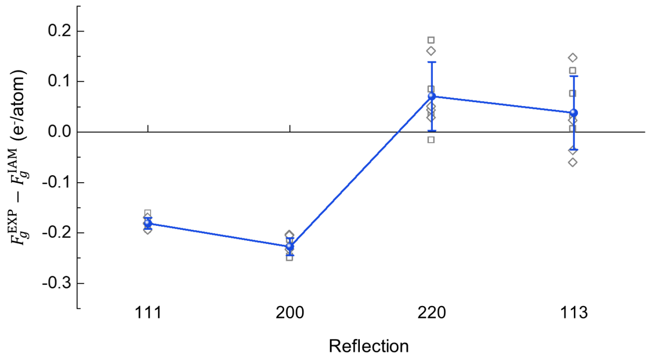

Supplementary Fig. 14 Difference between experimentally measured structure factors and IAM structure factors. F_{111} and F_{200} deviate from IAM values as all the measured data points are consistently on one side of the x-axis. For F_{220} and F_{113}, the data points are closer to the x-axis, and both of them are not different from the IAM values considering the error bar. Therefore, F_{220} and F_{113} may be influenced by bonding, but capturing minute deviation from IAM values for structure factors after (200) is difficult. The error bars represent the standard deviation calculated from 8 independent measurements for each data point.

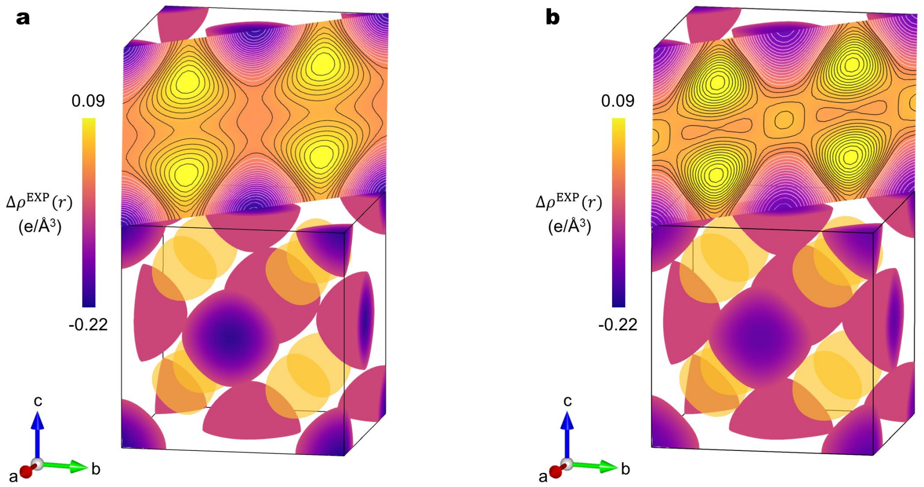

Supplementary Fig. 15 Deformation electron density (a) with and (b) without $\boldsymbol{F}_{220}$. In both cases, bonding electrons tend to accumulate at octahedral interstitial sites.

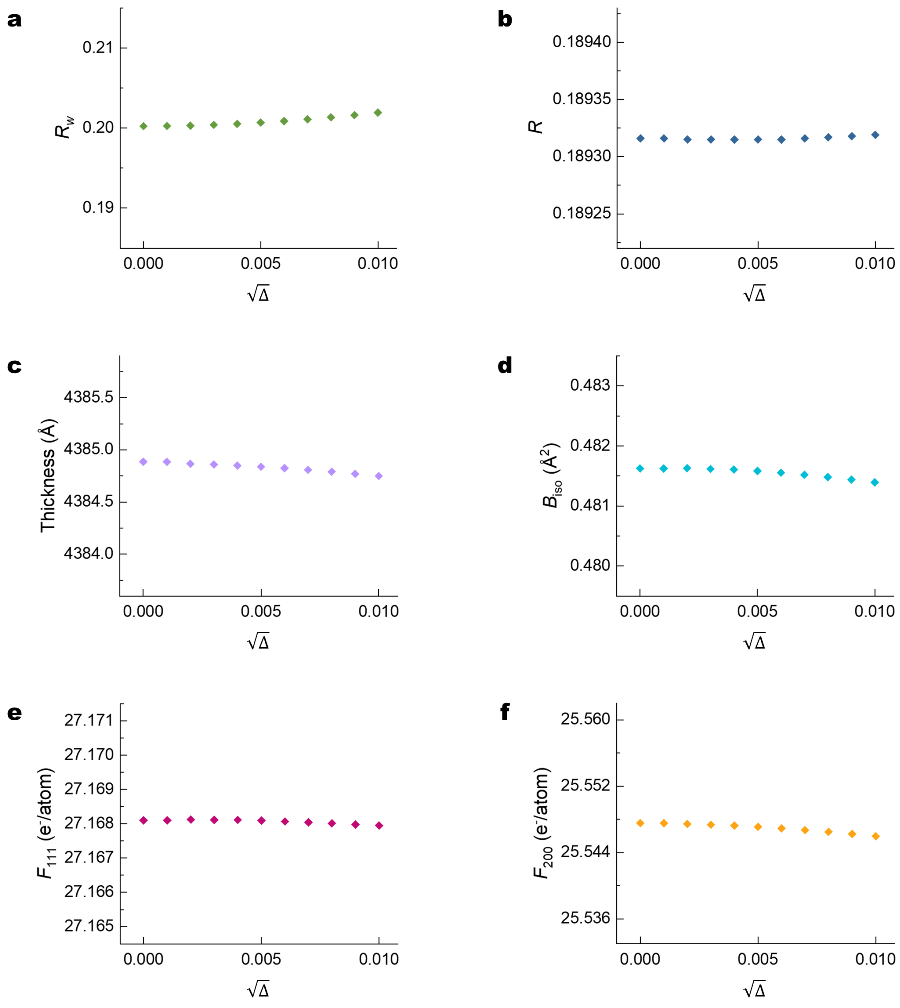

Supplementary Fig. 16 The refinement results as a function of linear gain. (a) The weighted R_w factor. **(b)** The standard R factor. **(c)** Thickness. **(d)** DWF. **(e, f)** F_{111} and F_{200}. DWF varies form 0.4814 Å^2 to 0.4816 Å^2 (a difference of 0.0002 Å^2). F_{111} varies from 27.1680 e$^-$/atom to 27.1681 e$^-$/atom (a difference of 0.0001 e$^-$/atom). F_{200} varies from 25.5460 e$^-$/atom to 25.5476 e$^-$/atom (a difference of 0.0016 e$^-$/atom).

Supplementary Table 1 Refined B_{iso} when different fitting parameters are relaxed during QCBED refinement

Image	Spot size (nm)	B_{iso}	B_{iso} F_{111}	B_{iso} F_{111} F_{200}	B_{iso} F_{111} F_{200} F_{220}	B_{iso} F_{111} F_{200} F_{220} F_{113}
1	0.5	0.667	0.581	0.514	0.507	0.505
2	0.5	0.552	0.472	0.430	0.435	0.380
3	0.5	0.592	0.541	0.485	0.471	0.447
4	0.5	0.653	0.611	0.505	0.490	0.492
Mean		0.616	0.551	0.483	0.476	0.456
STDEV		0.054	0.060	0.038	0.031	0.057
5	2.4	0.740	0.599	0.472	0.479	0.501
6	2.4	0.636	0.527	0.464	0.464	0.459
7	2.4	0.676	0.569	0.481	0.477	0.475
8	2.4	0.714	0.652	0.484	0.497	0.495
Mean		0.692	0.587	0.475	0.479	0.483
STDEV		0.045	0.052	0.009	0.013	0.019
Overall Mean		0.654	0.569	0.479	0.477	0.469
Overall STDEV		0.050	0.056	0.023	0.022	0.038

Supplementary Table 2 Refined F_{111}^{EXP} when different fitting parameters are relaxed during QCBED refinement

**Image**	**Spot size (nm)**	B_{iso}	B_{iso} F_{111}	B_{iso} F_{111} F_{200}	B_{iso} F_{111} F_{200} F_{220}	B_{iso} F_{111} F_{200} F_{220} F_{113}
1	0.5		27.272	27.182	27.203	27.216
2	0.5		27.253	27.168	27.162	27.226
3	0.5		27.261	27.182	27.194	27.215
4	0.5		27.304	27.200	27.248	27.264
Mean			27.273	27.183	27.202	27.230
STDEV			0.023	0.013	0.035	0.023
5	2.4		27.289	27.166	27.170	27.159
6	2.4		27.235	27.178	27.187	27.182
7	2.4		27.270	27.180	27.228	27.255
8	2.4		27.321	27.191	27.192	27.195
Mean			27.279	27.179	27.194	27.198
STDEV			0.036	0.010	0.024	0.041
Overall Mean			27.276	27.181	27.198	27.214
Overall STDEV			0.029	0.012	0.030	0.032

Supplementary Table 3 Refined F_{200}^{EXP} when different fitting parameters are relaxed during QCBED refinement

Image	Spot size (nm)	B_{iso}	B_{iso} F_{111}	B_{iso} F_{111} F_{200}	B_{iso} F_{111} F_{200} F_{220}	B_{iso} F_{111} F_{200} F_{220} F_{113}
1	0.5			25.502	25.496	25.529
2	0.5			25.520	25.518	25.516
3	0.5			25.535	25.538	25.530
4	0.5			25.499	25.485	25.539
Mean				25.514	25.509	25.528
STDEV				0.017	0.023	0.010
5	2.4			25.545	25.546	25.546
6	2.4			25.516	25.516	25.483
7	2.4			25.543	25.530	25.601
8	2.4			25.522	25.522	25.531
Mean				25.532	25.528	25.540
STDEV				0.015	0.013	0.048
Overall Mean				25.523	25.519	25.534
Overall STDEV				0.016	0.018	0.029

Supplementary Table 4 Refined F_{220}^{EXP} when different fitting parameters are relaxed during QCBED refinement

Image	Spot size (nm)	B_{iso}	B_{iso} F_{111}	B_{iso} F_{111} F_{200}	B_{iso} F_{111} F_{200} F_{220}	B_{iso} F_{111} F_{200} F_{220} F_{113}
1	0.5				21.457	21.465
2	0.5				21.356	21.555
3	0.5				21.410	21.485
4	0.5				21.555	21.555
Mean					21.444	21.515
STDEV					0.084	0.047
5	2.4				21.415	21.374
6	2.4				21.423	21.420
7	2.4				21.533	21.542
8	2.4				21.400	21.402
Mean					21.443	21.434
STDEV					0.061	0.074
Overall Mean					21.444	21.475
Overall STDEV					0.073	0.061

Supplementary Table 5 The imaginary part of the structure factors from the literature and from QCBED refinement

	U_{111}	U_{200}	U_{220}
Bird & King	0.00556	0.00543	0.00500
EXP	0.00652	0.00680	0.00515

Supplementary Table 6 Difference between experimental and IAM electron diffraction structure factors

Image	Spot size (nm)	F_{111}^{EXP}-F_{111}^{IAM}	F_{200}^{EXP}-F_{200}^{IAM}	F_{220}^{EXP}-F_{220}^{IAM}	F_{113}^{EXP}-F_{113}^{IAM}
1	0.5	-0.180	-0.248	0.084	0.076
2	0.5	-0.194	-0.230	-0.017	0.033
3	0.5	-0.180	-0.215	0.037	0.006
4	0.5	-0.162	-0.251	0.182	0.121
Mean		-0.179	-0.236	0.072	0.059
STDEV		0.013	0.017	0.084	0.051
5	2.4	-0.196	-0.205	0.043	-0.038
6	2.4	-0.184	-0.234	0.050	-0.061
7	2.4	-0.182	-0.207	0.160	0.147
8	2.4	-0.171	-0.228	0.028	0.022
Mean		-0.183	-0.219	0.070	0.017
STDEV		0.010	0.015	0.061	0.093
Overall Mean		-0.181	-0.227	0.071	0.038
Overall STDEV		0.012	0.016	0.073	0.072

Supplementary Table 7 Low-order structure factors of tP2 unit cell for the construction of deformation electron density map

h	k	l	$\frac{\sin\theta}{\lambda}$	F_g^{DFT}	$\lvert F_g^{\mathrm{DFT}}\rvert$	Equivalent F_g^{DFT}
0	0	-1	0.131	-18.463	18.463	9.232
-1	0	0	0.186	-17.271	17.271	8.636
-1	0	-1	0.227	54.291	54.291	27.146
-1	-1	0	0.263	51.123	51.123	25.575
0	0	-2	0.263	51.178	51.178	

Supplementary Table 8 Low-order structure factors of oP4 unit cell for the construction of deformation electron density map

h	k	l	$\frac{\sin\theta}{\lambda}$	F_g^{DFT}	$\lvert F_g^{\mathrm{DFT}} \rvert$	Equivalent F_g^{DFT}
0	0	-1	0.093	27.115	27.115	6.779
0	-1	0	0.131	0.000	0.000	0.000
0	-1	-1	0.161	25.238	25.238	6.310
-1	0	0	0.186	0.000	0.000	0.000
0	0	-2	0.186	0.016	0.016	0.004
-1	0	-1	0.208	23.718	23.718	5.929
-1	-1	0	0.227	-108.597	108.597	27.151
0	-1	-2	0.227	108.610	108.610	
-1	-1	-1	0.246	-22.486	22.486	5.621
-1	0	-2	0.263	102.297	102.297	25.575
0	-2	0	0.263	-102.300	102.300	

Supplementary Table 9 Low-order structure factors of tP4 unit cell for the construction of deformation electron density map

h	k	l	$\frac{\sin\theta}{\lambda}$	F_g^{DFT}	$\lvert F_g^{DFT} \rvert$	Equivalent F_g^{DFT}
0	0	-1	0.066	27.683	27.683	13.842
0	0	-2	0.131	0.011	0.011	0.003
-1	0	0	0.186	0.000	0.000	0.000
-1	0	-1	0.197	24.074	24.074	6.026
0	0	-3	0.197	-24.137	24.137	
-1	0	-2	0.227	108.598	108.598	27.149
-1	-1	0	0.263	-102.322	102.322	25.578
0	0	-4	0.263	-102.301	102.301	

Supplementary Table 10 Low-order structure factors of tP8 unit cell for the construction of deformation electron density map

h	k	l	$\frac{\sin\theta}{\lambda}$	F_g^{DFT}	$\lvert F_g^{DFT} \rvert$	Equivalent F_g^{DFT}
-1	0	0	0.093	-38.345	38.345	4.793
-1	-1	0	0.131	0.024	0.024	0.002
0	0	-1	0.131	0.000	0.000	
-1	0	-1	0.161	35.701	35.701	4.463
-2	0	0	0.186	0.059	0.059	0.004
-1	-1	-1	0.186	0.000	0.000	
-2	-1	0	0.208	33.574	33.574	4.197
-2	0	-1	0.227	-217.184	217.184	27.148
-2	-1	-1	0.246	31.790	31.790	3.974
-2	-2	0	0.263	-204.622	204.622	25.576
0	0	-2	0.263	204.592	204.592	

Supplementary Table 11 Low-order structure factors of oP8 unit cell for the construction of deformation electron density map

h	k	l	$\frac{\sin\theta}{\lambda}$	F_g^{DFT}	$\lvert F_g^{\mathrm{DFT}} \rvert$	Equivalent F_g^{DFT}
0	-1	1	0.093	-19.179	19.179	2.397
0	-1	-1	0.093	-19.179	19.179	
-1	0	0	0.131	-36.932	36.932	3.464
0	-2	0	0.131	0.039	0.039	
0	0	2	0.131	36.942	36.942	
0	0	-2	0.131	36.942	36.942	
-1	-1	1	0.161	-17.832	17.832	2.229
-1	-1	-1	0.161	-17.832	17.832	
-1	-2	0	0.186	34.565	34.565	2.593
-1	0	2	0.186	0.025	0.025	
-1	0	-2	0.186	0.025	0.025	
0	-2	2	0.186	-34.558	34.558	
0	-2	-2	0.186	-34.558	34.558	
0	-3	1	0.208	-16.797	16.797	2.100
0	-1	3	0.208	-16.807	16.807	
0	-3	-1	0.208	-16.797	16.797	
0	-1	-3	0.208	-16.807	16.807	
-1	-2	2	0.227	217.184	217.184	27.148
-1	-2	-2	0.227	217.184	217.184	
-1	-3	1	0.246	-15.893	15.893	1.988
-1	-1	3	0.246	-15.917	15.917	
-1	-3	-1	0.246	-15.893	15.893	
-1	-1	-3	0.246	-15.917	15.917	
-2	0	0	0.263	204.580	204.580	25.572
0	-4	0	0.263	204.528	204.528	
0	0	4	0.263	204.602	204.602	
0	0	-4	0.263	204.602	204.602	

Supplementary Table 12 Detailed bonding strength between the nearest neighbours for different atom pairs

Configuration	Bond type	Number of data points	Electron density (e/Å^3)	Average (e/Å^3)	Standard deviation
tP2	Pd-Pd	1	0.3383	0.3383	0
	Pd-Fe	1	0.2724	0.2724	0
	Fe-Fe	1	0.2459	0.2459	0
oP4	Pd-Pd	2	0.3343<br>0.3298	0.33205	0.00318
	Pd-Fe	3	0.2732<br>0.2724<br>0.2676	0.27107	0.00303
	Fe-Fe	2	0.2465<br>0.2442	0.24535	0.00163
tP4	Pd-Pd	2	0.3352<br>0.3263	0.33075	0.00629
	Pd-Fe	1	0.2702	0.2702	0
	Fe-Fe	2	0.2453<br>0.2437	0.2445	0.00113
tP8	Pd-Pd	3	0.3354<br>0.3349<br>0.3298	0.33337	0.0031
	Pd-Fe	2	0.2744<br>0.2699	0.27215	0.00318
	Fe-Fe	3	0.2522<br>0.2427<br>0.2336	0.24283	0.0093
oP8	Pd-Pd	4	0.3368<br>0.3367<br>0.3342<br>0.3306	0.33458	0.00291
	Pd-Fe	9	0.2783<br>0.2763<br>0.2757	0.27321	0.00301

		0.2732		
		0.2723		
		0.2722		
		0.2718		
		0.2698		
		0.2693		
Fe-Fe	4	0.2478	0.24408	0.00346
		0.2462		
		0.2416		
		0.2407		

References

1. Bremer F., Beyss M., Wenzl H. The order–disorder transition of the intermetallic phase Ni3Al. *Physica status solidi (a)* **110**, 77-82 (1988).
2. Olivero J. J., Longbothum R. Empirical fits to the Voigt line width: A brief review. *J. Quant. Spectrosc. Radiat. Transfer* **17**, 233-236 (1977).
3. Egerton R., Li P., Malac M. Radiation damage in the TEM and SEM. *Micron* **35**, 399-409 (2004).
4. Wei J., *et al.* Direct imaging of atomistic grain boundary migration. *Nat. Mater.* **20**, 951-955 (2021).
5. Urban K. Radiation-Induced Processes in Experiments Carried out In-situ in the High-Voltage Electron Microscope. *Physica status solidi (a)* **56**, 157-168 (1979).
6. Nagase T., *et al.* MeV electron irradiation induced crystallization in metallic glasses: Atomic structure, crystallization mechanism and stability of an amorphous phase under the irradiation. *J. Non-Cryst. Solids* **358**, 502-518 (2012).
7. Burzo E., Vlaic P. Magnetic properties of iron-palladium solid solutions and compounds. *J. Optoelectron. Adv. Mater.* **12**, 1869-1878 (2010).
